# The Quantitative Measurement of Peptidoglycan Components Obtained from Acidic Hydrolysis in Gram-Positive and Gram-Negative Bacteria via Hydrophilic Interaction Liquid Chromatography Coupled with Mass Spectrometry

**DOI:** 10.3390/microorganisms11092134

**Published:** 2023-08-23

**Authors:** Dmitri Pismennõi, Anna Kattel, Isma Belouah, Ranno Nahku, Raivo Vilu, Eeva-Gerda Kobrin

**Affiliations:** 1 Center of Food and Fermentation Technologies (TFTAK), Mäealuse 2/4, 12618 Tallinn, Estonia; dmitri@tftak.eu (D.P.); anna.kattel@tftak.eu (A.K.); isma.belouah@tftak.eu (I.B.); ranno@tftak.eu (R.N.); raivo@tftak.eu (R.V.); 2 Department of Chemistry and Biotechnology, Tallinn University of Technology, Akadeemia Tee 15, 12618 Tallinn, Estonia

**Keywords:** peptidoglycan, HILIC-MS, hydrolysis, n-acetylmuramic acid, n-acetylglucosamine, muramic acid, glucosamine, biomass composition analysis

## Abstract

The high throughput in genome sequencing and metabolic model (MM) reconstruction has democratised bioinformatics approaches such as flux balance analysis. Fluxes’ prediction accuracy greatly relates to the deepness of the MM curation for a specific organism starting from the cell composition. One component is the cell wall, which is a functional barrier (cell shape, exchanges) with the environment. The bacterial cell wall (BCW), including its thickness, structure, and composition, has been extensively studied in *Escherichia coli* but poorly described for other organisms. The peptidoglycan (PG) layer composing the BCW is usually thinner in Gram− bacteria than in Gram+ bacteria. In both bacteria groups, PG is a polymeric mesh-like structure of amino acids and sugars, including N-acetylglucosamine, N-acetylmuramic acid, and amino acids. In this study, we propose a high-throughput method to characterise and quantify PG in Gram-positive and Gram-negative bacteria using acidic hydrolysis and hydrophilic interaction liquid chromatography coupled with mass spectrometry (HILIC-MS). The method showed a relatively short time frame (11 min analytical run), low inter- and intraday variability (3.2% and 4%, respectively), and high sensitivity and selectivity (limits of quantification in the sub mg/L range). The method was successfully applied on two Gram-negative bacteria (*Escherichia coli* K12 MG1655, *Bacteroides thetaiotaomicron* DSM 2079) and one Gram-positive bacterium (*Streptococcus salivarius* ssp. *thermophilus* DSM20259). The PG concentration ranged from 1.6% *w*/*w* to 14% *w*/*w* of the dry cell weight. The results were in good correlation with previously published results. With further development, the PG concentration provided by this newly developed method could reinforce the curation of MM.

## 1. Introduction

Peptidoglycan (PG) is a component of the bacterial cell wall involved in both the cell shape [1] and the resistance to osmotic stress. The PG layer is a mesh-like structure constantly synthesised, remodelled, and repaired to adjust to changes in the cell environment and physiology (division, sporulation). This mesh is composed of alternating units of N-acetylglucosamine (NAG) and N-acetylmuramic acids (NAM), connected with β-1-4 bonds and adjoined in long strands. The PG synthesis starts in the cytoplasm with the formation of glucosamine-6-phosphate from fructose-6-phosphate. After six more enzymatic reactions, an NAG residue is linked to an NAM, which bears a peptide stem consisting of five amino acids. This monomeric disaccharide peptide unit is called lipid II. During the polymerisation of PG, several units of lipid II are linked to each other via a short peptide bridge. The length of the PG strands, the composition of the disaccharide peptide units [2], and the thickness of the mesh [3] vary between strains [4]. Based on the PG KEGG pathway map (map00550), the synthesis of lipid II could cost up to seven ATP molecules, one UTP molecule, and one NAD(P)+ molecule with glucose as a carbon source [5]. In addition, one acetyl-Coa molecule, one phosphoenolpyruvate molecule, and one undecaprenyl phosphate molecule are mobilised. Most ATP is consumed during the extension of the peptide stem with one ATP molecule per added amino acid. The PG layer is thus an energy and amino acids sink that should be further characterised, especially for its application in in silico metabolic modelling analysis. In the absence of specific information, the biomass composition is usually derived from the closest well-known organism, such as *E. coli* or *B. subtilis*. Improving the specificity and stoichiometry of the biomass reaction within models would certainly reinforce the accuracy of the predicted fluxes [6,7]. However, to keep up with the rapid pace of in silico approaches, the methods for biomass characterisation must be constantly improved.

The analysis of PG is performed in two principal ways: a qualitative description of PG in a selected strain of bacteria [8,9,10,11] or a quantitative measurement of selected biomarkers, e.g., muramic acid (Mur) [12,13] or meso-diaminopimelic acid (mDAP) [14]. The typical approach to obtain quantitative results from measurements of selected biomarkers from PG is to perform acidic hydrolysis of biomass to release those biomarkers in their free unbound form, making them detectable via various analytical techniques [15]. Liquid chromatography coupled with mass spectrometry is the most well-known technique for unambiguously determining peptidoglycan layer components or their structure. Depending on the type of the instrument, quantitative and qualitative data could be obtained through various sample preparation methodologies reported in the literature [13,16]. Due to the nature of NAM and NAG, they are usually derivatised to improve their volatility for gas chromatographs with mass spectrom·etery analysis [17] or reduced via strong bases to eliminate anomeric centres [8]. However, the high complexity of sample preparation methods raises the risk of introducing potential contaminants and increasing the sample turnover time accordingly. Therefore, the present work aims to introduce a simple, rapid, and reliable methodology for quantitative measurements of N-acetylmuramic acid, N-acetylglucosamine, and products of their acidic hydrolysis via hydrophilic interaction liquid chromatography coupled with mass spectrometry.

## 2. Materials and Methods

### 2.1. Chemicals and Materials

The standards for the components of the peptidoglycan layer, including N-acetylmuramic acid (NAM), N-acetylglucosamine (NAG), muramic acid (Mur), and glucosamine hydrochloride (GlcN), were obtained from Sigma-Aldrich (Darmstadt, Germany). Glucosamine-^13^C_6_ hydrochloride (GlcN-C13, UL-^13^C_6_, 99% ^13^C enrichment) was procured from Omicron Biochemicals, Inc. (South Bend, IN, USA). Ammonium acetate (AmAc, HiPerSolv CHROMANORM^®^ for LC-MS), ammonium formate (AmFor, HiPerSolv CHROMANORM^®^ for LC-MS), and acetic acid (AA, HiPerSolv CHROMANORM^®^ for LC-MS) were purchased from VWR International GmbH (Wien, Austria). Acetonitrile (MeCN; LiChrosolv, HPLC gradient grade), isopropanol (IPA; LiChrosolv, HPLC gradient grade), formic acid (FA, LC-MS grade), diethylamine (DEA, ≤99% purity), phenol (ACS reagent, 99.0–100.5% purity), hydrochloric acid (HCl, 36.5–38% purity), guanidine chloride (GuHCl, ≤99% purity), and ammonium hydroxide (25%, LC-MS LiChropur™ grade) were procured from Honeywell (Charlotte, NC, USA). Ultrapure water (≤18.2 MΩ·cm) was produced in-house with MilliQ^®^ HX7040SD equipped with MilliQ LC-PAK (Merck KGaA, Darmstadt, Germany). Biotage Isolute^®^ PLD+ columns were bought from Biotage Sweden AB (Uppsala, Sweden).

### 2.2. Preparation of Standard Solutions

The stock solutions of individual peptidoglycan layers’ components were prepared firstly in ultrapure water and stored at −20 °C. The solution of the isotopically labelled standard was prepared in ultrapure water and stored at −50 °C. The working solutions of both non-labelled and labelled standards were prepared via dilution in 100% MeCN and then serially diluted with 50% aqueous MeCN (*v*/*v*). Calibration curves were built for N-acetylmuramic acid (0.66–42.50 mg/L), N-acetylglucosamine (1.58–50.50 mg/L), muramic acid (0.63–40.85 mg/L), and glucosamine (0.80–52.25 mg/L). The internal standard was added to the autosampler vial before the injection, and its concentration was fixed at 6.81 mg/L. The calibration curves were built using the response factor calculated according to Equation (1).

Response Factor = Area of analytes × (Concentration of internal standard/Area of internal standard)(1)

Calibration curves were built using seven-point measurements of serially diluted standard solutions. The regression was found by fitting points to a quadratic equation.

### 2.3. Liquid Chromatography

The standards and samples were analysed using a Waters Acquity H-Class Plus Bio UPLC^®^ system (Waters Corporation, Milford, MA, USA) coupled with a Waters Acquity QDa detector (Waters Corporation, Milford, MA, USA) controlled by Waters Empower 3 (Build 3471 FR5 SR4, Waters Corporation, Milford, MA, USA). The optimal mobile phases (MPs) were (MPA) 20 mM ammonium acetate in ultrapure water with adjusted pH = 4.75 ± 0.02 and (MPB) 90% MeCN + 10% 20 mM ammonium acetate in ultrapure water with adjusted pH = 4.75 ± 0.02. The wash solvent was 10% ultrapure water in MeCN (*v/v*), and the purge solvent composition was 10% MeCN in ultrapure water (*v/v*). The seal wash was 20% MeCN in ultrapure water (*v/v*). The standards and samples were stored in the autosampler compartment cooled to 8 °C. The injection volume was set to 3 µL. Several columns were tested: the Waters XBridge^®^ BEH Amide XP (3.0 × 150 mm, 2.5 µm, Waters Corporation, Milford, MA, USA), Waters Atlantis Premier BEH C18 AX (2.1 × 100 mm, 1.7 µm, Waters Corporation, Milford, MA, USA), Phenomenex Luna Omega Sugar column (2.1 × 150 mm, 100 Å, 3 µm, Phenomenex Inc., Torrance, CA, USA), Waters Acquity UPLC^®^ BEH Phenyl (2.1 × 100 mm, 1.7 µm, Waters Corporation, Milford, MA, USA), and Waters Atlantis Premier BEH Z-HILIC (2.1 × 150 mm, 1.7 µm, Waters Corporation, Milford, MA, USA). An ACQUITY UPLC Column in-line filter unit (Waters Corporation, Milford, MA, USA) with an installed 0.2 µm stainless steel filter was used in all experiments with all tested columns. The optimal column temperature was 60 °C for the duration of an analytical run. The optimised gradient was as follows: 0–1 min for 5% A, 1–3 min for linear gradient 5–55%, 3.01–5 min for linear gradient 55–60%, 5.01–7 min for a hold at 60% A, and 7.01–11 min for a hold at 5% A. The optimal flow rate for those experiments was found to be 500 µL/min.

### 2.4. Mass Spectrometry

The analytes were ionised under electrospray ionisation conditions with optimised source conditions. The source temperature was set at 120 °C, and high-purity nitrogen was fed into the source at 1200 L/h (desolvation) and heated to 600 °C. The optimisation was conducted by injecting a high concentration standard with varying parameters, such as the capillary voltage, cone voltage, and gas temperature, with the optimised gradient parameters described earlier. The capillary voltage was set at −0.8 kV, and the optimal cone voltage for all compounds was found at −5 V. Firstly, the measurements were conducted in the scan mode to find the appropriate adducts for each compound of interest. During these experiments, appropriate single-ion recording channels were selected for optimal detection and analyte identification. The chosen adducts and selected ion recording (SIR) channels corresponding to *m*/*z* can be found in Table 1:

Data were acquired and analysed in Waters Empower 3 (Build 3471 FR5 SR4, Waters Corporation, Milford, MA, USA). Additional data analysis was performed in Microsoft Excel (Microsoft 365 Apps for enterprise, Microsoft Corp., Redmond, WA, USA) and GraphPad Prism (v 9.0.0, GraphPad Software, LLC, Boston, MA, USA).

### 2.5. Bacterial Growth Description

All strains were cultivated at 37 °C, but the gas environment and media were modified according to species. *Escherichia coli* K12 MG1655 (ECO) was cultured aerobically on the shaker, set at 110 rpm (Ecotron, Infors HT, Switzerland). For ECO, modified LB-Luria broth was used, which contained (per litre) 10 g of casitone (Tryptone Plus, Fluka Analytical), 5 g of yeast extract (NuCell 545), 0.5 g of NaCl, 2.93 g of K_2_HPO_4_, and 4.65 g of KH_2_PO_4_.

*Streptococcus salivarius* ssp. *thermophilus* DSM 20259 (STH) was cultivated aerobically in the incubator in M-17 broth (Sigma-Aldrich). *Bacteroides thetaiotaomicron* DSM 2079 (BTH) was cultured in the thermostat inside the anaerobic chamber (COY box, Coy Laboratory Products Inc., Grass Lake, MI, USA) with an atmosphere of 2.5 ± 0.5% H_2_, 10% CO_2_, and balanced N_2_. The medium composition was as follows (per litre): 5 g of D-glucose, 2.5 g of casitone (Tryptone Plus, Fluka Analytical), 2.5 g of yeast extract (NuCell 545), 1 g of L-cysteine HCl, 2.93 g of K_2_HPO_4_, 4.65 g of KH_2_PO_4_, 0.9 g of (NH_4_)_2_SO_4_, 0.9 g of NaCl, 0.28 g of KOH, 1 g of NaHCO_3_, 10 mg of hemin, 10 µg of biotin, 10 µg of cobalamin, 30 µg of para-aminobenzoic acid, 150 µg of pyridoxamine, 50 µg of folic acid, 0.09 g of MgSO_4_·7 H_2_O, and 0.09 g of CaCl_2_·2 H_2_O.

### 2.6. Sample Preparation

After growth, all cultures were washed with a saline solution to eliminate media components interfering with the analytical measurements and concentrated 20 times to reach higher biomass densities. After concentrating, cultures were washed again with ultrapure water for further purification. A washed bacterial culture was then aliquoted into smaller portions and frozen at −50 °C until analysis. Analysed aliquots were thawed at room temperature. The thawed bacterial culture was transferred to a 10 mL volumetric flask and filled with ultrapure water up to the mark. The dissolved bacterial culture was then freeze-dried in a 2 mL Eppendorf vial. The dried bacterial culture was then subjected to acidic hydrolysis. The hydrolysis was performed with a mixture containing 6N hydrochloric acid (HCl) with 1% phenol (*v/v*) in an Eppendorf Thermomixer^®^ C thermomixer (Eppendorf AG, Hamburg, Germany) with a temperature set at 100 °C and stirring at 1000 rpm. The hydrolysis was carried out for 4 h. As samples contained a large quantity of HCl and phenol, the mixture of HCl and phenol was removed with vacuum centrifugal evaporation. Dried hydrolysed bacterial cultures were resuspended in 50% aqueous MeCN (*v/v*) before the clean-up with Biotage PLD+ cartridges. Samples were loaded on Biotage PLD+ cartridges and eluted in vacuo to ensure clean extraction. The extracts were diluted accordingly with 50% aqueous MeCN (*v/v*), and, as the last step, the isotopically labelled internal standard was added to the autosampler vial.

### 2.7. Method Validation

The developed method was assessed for linearity (as a correlation coefficient of R^2^ of the calibration curve), the limit of detection and quantification (as the standard deviation of the measured sample at the lowest calibration points multiplied by 3 or 10, respectively), and the recovery of analytes during the sample preparation steps [18].

## 3. Results

### 3.1. Optimisation of Chromatographic and Mass Spectrometric Parameters

**Figure 1:**
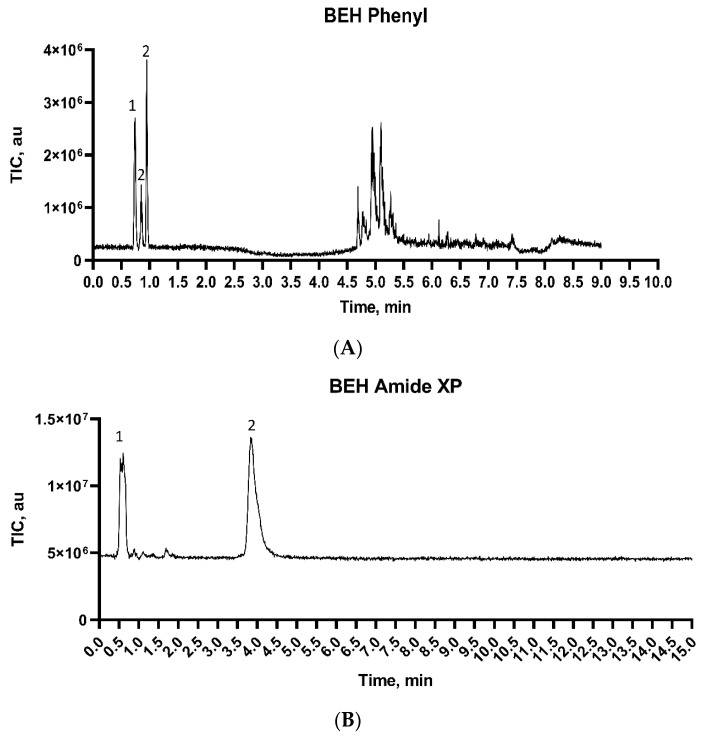

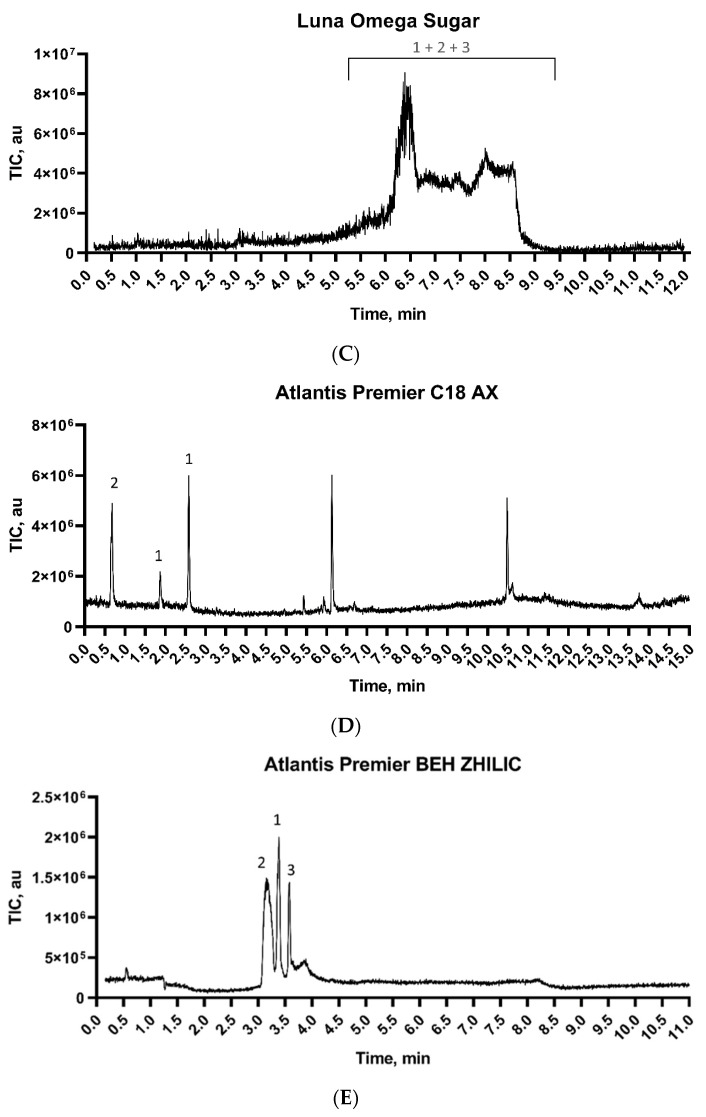
(**A**) Waters Acquity BEH Phenyl column (2.1 × 100 mm, 1.7 µm). Mobile phases were (MPA) 0.1% FA in ultrapure water and (MPB) MeOH. Gradient elution was used, and the flow rate was 300 µL/min. (**B**) Waters XBridge BEH Amide XP (3.0 × 150 mm, 2.5 µm). Mobile phases were (MPA) 80/20/0.05 MeCN/ultrapure water/DEA + 0.5 mg/L GuHCl and (MPB) (**A**) 90/5/5/0.05 MeCN/ultrapure water/isopropanol/DEA + 0.5 mg/L GuHCl. The gradient elution program was used, and the flow rate was 800 µL/min. The methodology was adapted from reference [19]. (**C**) Phenomenex Luna Omega Sugar (2.1 × 150 mm, 3 µm). The mobile phases were (MPA) 100 ultrapure water + 0.5 mg/L GuHCl and (MPB) 99/1 MeCN/ultrapure water + 0.5 mg/L GuHCl. The gradient elution program was used, and the flow rate was 313 µL/min. The methodology was adapted from reference [20]. (**D**) Waters Atlantis Premier BEH C18 AX (2.1 × 100 mm, 1.7 µm). The mobile phases were (MPA) 10 mM of AmFor in ultrapure water with pH = 3.75 and (MPB) 90/10 MeCN/10 mM AmFor in ultrapure water with pH = 3.75. The gradient elution program was used, and the flow was set to 300 µL/min. (**E**) Waters Atlantis Premier BEH Z-HILIC (2.1 × 150 mm, 1.7 µm). The mobile phases were (MPA) 20 mM of AmAc in ultrapure water with pH = 4.75 and (MPB) 90/10 MeCN/20 mM AmAc in ultrapure water with pH = 4.75. The gradient elution program was used, and the flow rate was set to 500 µL/min.

Our first experiments were performed by injecting only NAM and NAG as primary components of any PG layer in Gram-positive and Gram-negative bacteria. Five different columns were selected and tested based on the nature of the analytes of interest. The chromatogram obtained for each of the five columns is reported in Figure 1. The performances of the columns were evaluated based on the capability to separate the different PG monomers: (1) NAG, (2) NAM, (3) Mur, and (4) GlcN.

**Figure 2:**
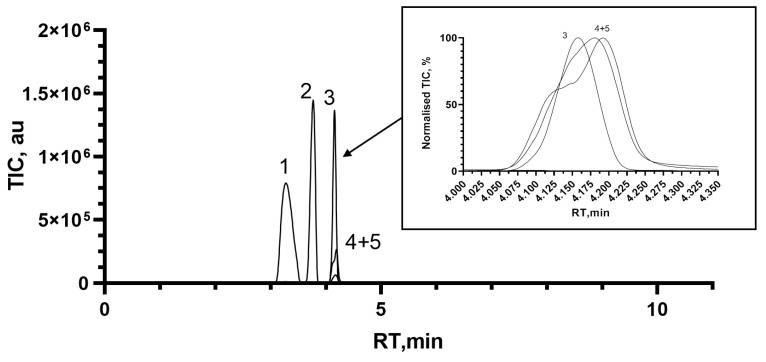
The optimised separation of NAG (1), NAM (2), Mur (3), GlcN (4), and GlcN-^13^C_6_ (5) obtained on Waters Atlantis Premier BEH Z-HILIC column in SIR experiments. The zoomed part is shown in normalised TIC levels.

During the development of a liquid chromatographic method, it was observed that most of the tested columns could not either retain the compounds of interest or present peak splitting for analytes of interest due to the formation of anomers. Waters Atlantis Premier BEH Z-HILIC was the most efficient out of five columns in terms of the separation power and retention of analytes on the column. A high column temperature and fast flow of mobile phases were applied to improve peak shapes and reduce anomers’ formation during the analytical run. The chromatograms obtained with the final optimised method are provided in Figure 2.

Due to the presence of a trace amount of chloride ions in the mobile phases, a chloride adduct formation for NAG and GlcN was observed, which allowed for more precise and cleaner spectra for those analytes [21]. However, NAM and Mur’s adduct formation had not been driven by the chloride ions in the mobile phases and was measured as deprotonated adducts. The reproducibility of adduct formation was closely monitored, and the repeatability of recorded response factors was very high. The gas flow and capillary voltages were set according to the manufacturer’s recommendations for operation at the high flow of mobile phases.

### 3.2. Methodology Validation

The optimised LC-MS methodology was validated, and the results are presented in Table 2.

Additionally, the methodology was controlled for the repeatability of retention times (RTs) and peak areas in standards over several independent experiments by injecting a standard mixture (Table 3).

### 3.3. Optimisation of Sample Preparation Procedures and Measurements of Bacterial Biomass

**Figure A1:**
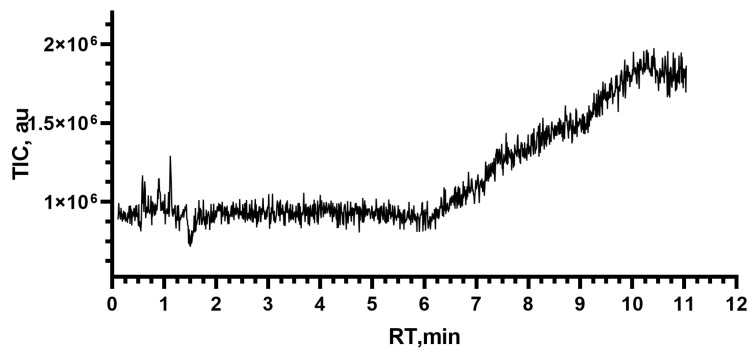
The LC-MS chromatogram obtained after lysozyme application on STH WBM.

Based on the literature on breaking down the bacterial wall with enzymes [22], the first experiments were conducted with a STH bacterial culture, which should possess a thick PG layer. The wet biomass (WBM) was aliquoted in several Eppendorf tubes and subjected to hydrolysis via a lysozyme solution according to a protocol published by Sigma-Aldrich (Merck KGaA, Darmstadt, Germany) [23]. The first results provided by this approach showed that the PG layer could not be hydrolysed into individual components, and, overall, no PG components were detected (Figure A1).

The current method was further optimised by improving the cell wall hydrolysis based on procedures described and adapted in the literature [24,25,26]. Reported methods tend to require very harsh and complex conditions, such as an oxygen-free environment, a high load of HCl acid to sample amount, and significant processing times due to the manual labour involved in preparing fully enclosed glass vessels containing both sample and hydrolysis reagents. The scaled-down methodology was tested to minimise processing times and decrease sample turnaround times. The amount of dried sample was reduced to *ca* 1–2 mg of dried biomass (DBM) and mixed with 300 µL of 6 M HCl containing 1% phenol (*v/v*). Furthermore, the samples were agitated for 4 h at 1000 rpm to improve the hydrolysis, and the temperature was reduced to 100 °C from the commonly employed 110–115 °C.

Additionally, the maintenance of the complete integrity of the standards after the acidic hydrolysis was used for WBM was evaluated. The hydrolysis was performed using an aliquot of a standard stock solution (*ca* 1 mg/mL). HCl with 1% phenol was evaporated in vacuo, and the residue was redissolved in 50% aqueous MeCN (*v/v*). This mixture was passed through a Biotage PLD+ cartridge to remove impurities from sample matrices. The filtrate was diluted with an internal isotopically labelled standard and injected on LC-MS as described in Section 2.3 of the Section 2. The recovery values for analytes were calculated and are presented in Table 4.

**Figure A2:**
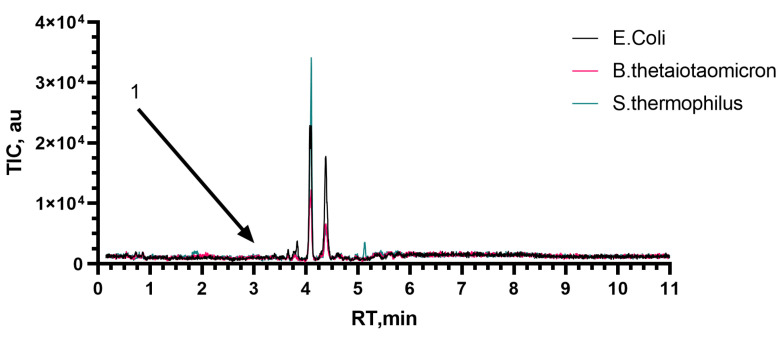
The LC-MS chromatogram of NAG (1) obtained during 4 h of acidic hydrolysis.

**Figure A3:**
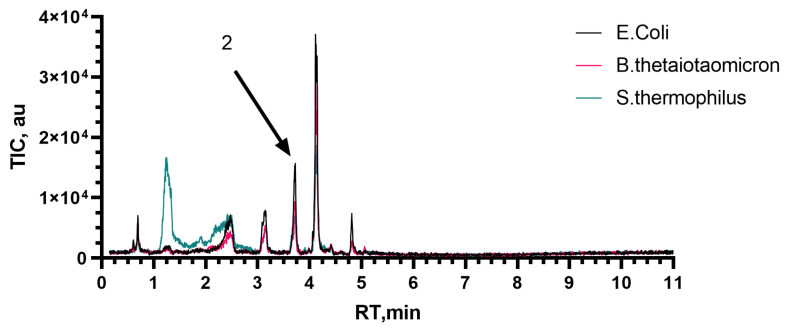
The LC-MS chromatogram of NAM (2) obtained during 4 h of acidic hydrolysis.

**Figure A4:**
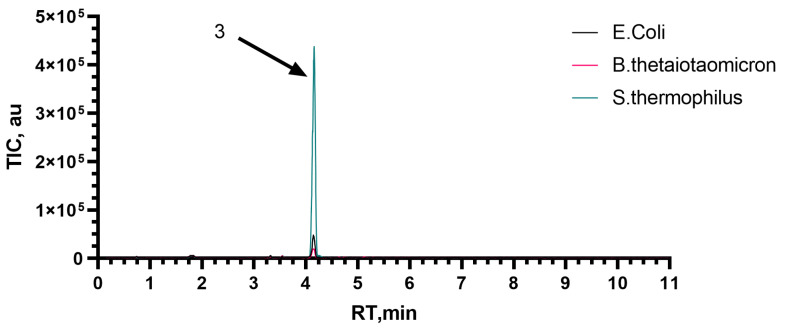
The LC-MS chromatogram of GlcN (3) obtained during 4 h of acidic hydrolysis.

**Figure A5:**
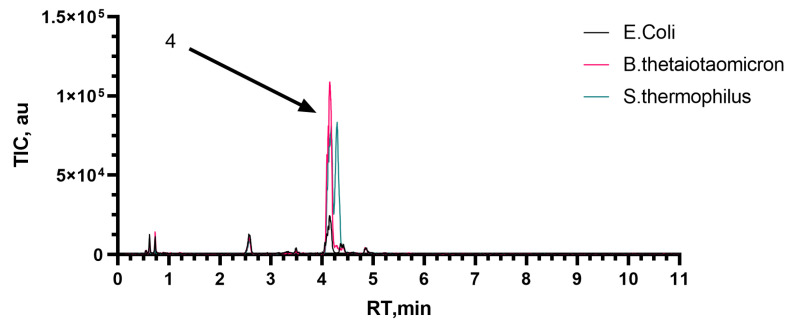
The LC-MS chromatogram of Mur (4) obtained during 4 h of acidic hydrolysis.

The hydrolysis reaction time was screened based on the Gram-staining of selected bacteria. In the case of Gram-negative bacteria, hydrolysis was conducted for four hours, sampling at 0.5, 1, 1.5, 2, and 4 h. For Gram-positive bacteria, the hydrolysis reaction time was prolonged to 16 h with sampling at 1.5, 2, 4, 12, and 16 h. All experiments were performed in triplicate, and an example chromatogram is shown in the Appendix A Section (Figure A2, Figure A3, Figure A4 and Figure A5). The percentage of analyte mass concentration per 1 mg of dry cell weight (*w/w*) is reported in Table 5 and Table 6. 

For Gram-negative bacteria, the release of PG components has increased with prolonged hydrolysis times, reaching its maximum concentrations by the 4th hour. However, for Gram-positive bacteria, longer hydrolysis times resulted in a slight decrease in quantified PG components with more significant experimental errors between replicates. Under optimised hydrolysis conditions, the recovery of PG’s hydrolysis products was 94 to 99% (Table 4), whereby GlcN showed a slight sign of degradation under used conditions. For Gram-positive bacteria, the hydrolysis time might be reduced to 2 h while maintaining high yields of PG components with low experimental errors. This behaviour could be attributed to structural differences between Gram-positive and Gram-negative bacteria in terms of an outer layer of the cell wall. In Gram-negative bacteria, the outer layers are the lipopolysaccharide layer and outer membrane, which might reduce the hydrolysis reaction rate.

## 4. Discussion

Acidic hydrolysis is a very viable and proven methodological approach to breaking down the rigid structures of the bacterial cell independent of the thickness of the cell wall. 

The results showed that, for widely studied microorganisms such as *Escherichia coli* K12 (ECO), the PG layer components’ concentration was in the range of 1.7% of the sum of amino sugars components per 1 mg of dry cell weight (Table 5). This value is in good agreement with previously published results on *Escherichia coli* B/r [27], which shows methodology robustness towards Gram-negative bacteria. Additionally, the PG concentration in BTH was at levels exceeding 3.6% (Table 5). According to a previously published study, the concentration of PG in BTH was calculated within the range of 1.1% to 1.3% [28]. As the PG layer comprises stoichiometrically equal amounts of both NAG and NAM, it was hypothesised that BTH could hold extra amounts of UDP-NAG as an intermediate of the cell wall synthesis. Additionally, it has been reported that, in *Escherichia coli* K-12 strain NCM3722, UDP-NAG is stored in the cytosol for synthesising the cell wall in case of changes in the growth environment, which could also be the case with BTH [29]. If PG components for BTH are calculated based on the stoichiometry principle, the sum of PG components for BTH would be in a proposed range of 1.14% of PG components per 1 mg of dry cell weight, which is in good agreement with calculated values of PG concentration from the literature. As for Gram-positive bacteria, STH showed a significantly higher concentration of PG components than Gram-negative ones (Table 6). There was no reported value for PG % in cell walls of STH, but for other Gram-positive bacteria, such as from the *Lactobacillales* order, the values for hexosamines, e.g., NAG and NAM, are in the range of 5.2–12.2% [30]. This range indicates that STH’s recorded sum of amino sugars of 8.2% is in good agreement with other similar Gram-positive bacteria, which possess a thick peptidoglycan layer. The greater concentration of NAG in STH compared to NAM could be attributed to the presence of UDP-NAG in the cytosol, as discussed previously.

## 5. Conclusions

With the developed HILIC-MS methodology described in this study, four different PG components could be accurately and repeatably quantified from the cell walls of both Gram-positive and Gram-negative bacteria. Although requiring sophisticated equipment, the HILIC-MS methodology allows an accurate quantification from a small amount of biomass and small working volume. The turnaround time could be significantly reduced from hours to minutes by employing different heat sources, such as microwave-based irradiation, at the hydrolysis step. The HILIC-MS methodology can also be adapted for 96-well plates for a higher throughput. In addition, the composition in PG of more prokaryotes should be determined in further studies. Nonetheless, the results obtained combined with metabolome and proteome data constitute a valuable input for the MM.

## Figures and Tables

**Table 1:** The adducts and *m*/*z* values for analytes of interest.

No.	Analyte	Adduct	*m*/*z*
1	N-acetylmuramic acid	[M−H]^−^	292.1
2	N-acetylglucosamine	[M+Cl]^−^	256.1
3	Muramic acid	[M−H]^−^	250.1
4	Glucosamine	[M+Cl]^−^	214.1
5	Glucosamine-^13^C_6_	[M+Cl]^−^	220.1

**Table 2:** Regression equations, correlation coefficients (R^2^), limits of detection (LoD), and quantification (LoQ) for analytes of interest.

Analyte	Regression Equation	R^2^	LoD, mg/L	LoQ, mg/L
N-acetylmuramic acid	Y = −2.45 × 10^−2^ X^2^ + 4.83 × 10^0^ X − 3.10 × 10^0^	0.9947	0.116	0.386
N-acetylglucosamine	Y = −5.83 × 10^−2^X^2^ + 6.77 × 10^0^ X + 3.46 × 10^0^	0.9973	0.345	1.149
Muramic acid	Y = 1.78 × 10^−2^ X^2^ + 1.76 × 10^0^ X − 1.16 × 10^0^	0.9933	0.048	0.159
Glucosamine	Y = −7.96 × 10^−3^ X^2^ + 9.18 × 10^−1^ X − 1.33 × 10^−1^	0.9999	0.015	0.050

**Table 3:** The inter- and intraday repeatability of RT and peak area expressed as relative standard deviation (RSD), %.

Analyte	RT		Peak Area	
	Interday RSD, % (n = 3)	Intraday RSD, % (n = 4)	Interday RSD, % (n = 3)	Intraday RSD, % (n = 4)
N-acetylmuramic acid	0.11	0.15	1.82	3.63
N-acetylglucosamine	0.12	0.20	3.19	5.00
Muramic acid	0.09	0.14	1.54	2.62
Glucosamine	0.08	0.12	0.20	1.03
Glucosamine-^13^C_6_ ^a^	0.08	0.13	8.68	9.6

^a^ Internal standard for quantification.

**Table 4:** The recovery values for pure standards after acidic hydrolysis under conditions used for WBM.

Analyte	Recovery (%, n = 3)	SD (%, n = 3)
N-acetylmuramic acid	0	0
N-acetylglucosamine	0	0
Muramic acid	99.1	3.8
Glucosamine	94.2	1.9

**Table 5:** The results of acidic hydrolysis performed with DBM of ECO and BTH in triplicate (Gram-negative bacteria).

Sampling Time, h	%Glucosamine (ECO)	%Muramic Acid (ECO)	%Glucosamine (BTH)	%Muramic Acid (BTH)
0.5	0.50 ± 0.02	0.47 ± 0.07	3.22 ± 0.23	0.49 ± 0.06
1	0.50 ± 0.02	0.51 ± 0.04	3.16 ± 0.03	0.52 ± 0.04
1.5	0.50 ± 0.08	0.52 ± 0.05	3.16 ± 0.16	0.51 ± 0.05
2	0.60 ± 0.06	0.59 ± 0.08	3.09 ± 0.33	0.55 ± 0.05
4	0.82 ± 0.10	0.88 ± 0.05	3.05 ± 0.02	0.57 ± 0.03

**Table 6:** The results of acidic hydrolysis performed with DBM of STH in triplicate (Gram-positive bacteria).

Sampling Time, h	%Glucosamine	%Muramic Acid
1.5	4.63 ± 0.12	3.47 ± 0.07
2	4.88 ± 0.03	3.51 ± 0.04
4	4.72 ± 0.31	3.52 ± 0.17
12	4.73 ± 0.27	3.53 ± 0.28
16	4.49 ± 0.42	3.38 ± 0.25

## Data Availability

Data are contained within the article.
